# A mixed-methods systematic review investigating the use of digital health interventions to provide palliative and end-of-life care for patients in low- and middle-income countries

**DOI:** 10.1177/26323524241236965

**Published:** 2024-04-12

**Authors:** Weerasingha Navarathnage Sachintha Dilhani, Sarah Mitchell, Jeremy Dale, Kavanbir Toor, Mikail Javaid, John I. MacArtney

**Affiliations:** Ministry of Health, Sri Lanka; Division of Primary Care, Palliative Care and Public Health, University of Leeds, Leeds, UK; Unit of Academic Primary Care, Warwick Medical School, University of Warwick, Coventry, UK; Warwick Medical School, University of Warwick, Coventry, UK; Warwick Medical School, University of Warwick, Coventry, UK; Unit of Academic Primary Care, Warwick Medical School, University of Warwick, Medical School Building, Coventry, West Midlands CV4 7AL, UK

**Keywords:** digital health, e-health, end-of-life care, low-income countries, middle-income countries, mobile health, palliative care, telehealth, telemedicine

## Abstract

**Background:**

The need for palliative care is rising globally with 76% of those who are in need living in low- and middle-income countries (LMICs). Digital health interventions (DHIs) have been identified as a means of making palliative care more widely accessible. This review summarizes the range and characteristics of DHIs used to deliver palliative care in LMICs and sought to identify factors that influence their implementation and utilization.

**Objectives:**

This review aims to summarize the range and characteristics of DHIs used to deliver palliative care in LMICs and identify factors that influence their implementation and utilization.

**Design:**

Mixed-method systematic review incorporating both quantitative and qualitative data.

**Data sources and methods:**

All studies focusing on DHIs for patients who need palliative care (adults/children) and their caregivers (patient and caregiver centred) in LMICs and published in English were identified through a systematic search of MEDLINE, EMBASE, PsycINFO and CINAHL databases. Data synthesis and analysis were carried out following the convergent integrated approach based on the Joanna Briggs Institute (JBI) methodology for mixed-methods systematic reviews.

**Results:**

Fifteen studies were included (three qualitative, four mixed-methods and eight quantitative studies). Telemedicine/mHealth was the most reported DHI utilized in LMICs in delivering palliative care. Patients and caregivers benefited from using DHIs in many ways including increased access to care with reduced discomfort, travel time and risk of health care-associated infections. Health care providers also reported that using DHI such as telemedicine enables them to provide care in a more effective and efficient way. Four factors were identified as the main barriers to implementation: resource constraints; literacy, training and skills; governance, operational and communication issues and technical issues.

**Conclusion:**

DHIs, such as telemedicine, have the potential to enhance accessibility to palliative care in LMICs, particularly in rural areas. Comprehensive strategies for their use are required to address the identified barriers.

## Introduction

Palliative care has been recognized worldwide as a component of comprehensive services required for managing serious health-related suffering across all conditions including cardiovascular diseases, cancer, chronic respiratory diseases, AIDS, diabetes and organ failure.^1,2^ It is an approach that seeks to improve the quality of life of patients and their families facing a life-threatening illness,^1,2^ through early identification, correct assessment and treatment of pain and other physical, psychological, social or spiritual problems.^1^

The need for palliative care is rising globally due to increases in the ageing population and non-communicable diseases.^2^ The estimated number of people with palliative care needs was over 56.8 million globally in 2020, of whom 76% live in low- and middle-income countries (LMICs)^2^ with a Gross National Income (GNI) per capita of between $1045 and $12,695 per annum.^3^ However, palliative care is not available to most people in need, especially in LMICs.^2,4–6^ There are various barriers including lack of clear policies establishing palliative care, lack of basic, intermediate and specialist training programmes in palliative care and inadequate training for the wider hospital and primary care workforce, limited physical infrastructure, lack of essential medications and lack of research in palliative care in LMICs.^2,4,5^ In many regions, particularly in remote or rural areas, access to specialized health facilities and restricted access to adequate medical–technical care are additional challenges^7,8^ in delivering palliative care. Furthermore, most people in LMICs who need palliative care stay at home and cannot easily travel beyond their communities.^4^ It is therefore important to ensure that palliative care is accessible in the home setting, and community.^4^

Digital health is the use of information and communications technologies in health professions to manage illnesses and to promote wellness, encompassing e-health (which includes mHealth), telehealth and telemedicine, as well as emerging areas, such as the use of advanced computing sciences in ‘big data’, genomics and artificial intelligence.^9,10^ In recent years, digital health interventions (DHIs) have been recognized as a facilitator in efforts to address the challenges limiting universal health coverage.^9^ DHIs were used increasingly in the delivery of palliative care during the COVID-19 crisis, mainly to deliver clinical and supportive interventions at a distance.^11–14^ The term digital health and other related terms are defined in Table 1.

**Table 1.** Definitions of terms related to digital health interventions.

Term	Definition
Digital health	A broad umbrella term encompassing e-health (which includes mHealth), health information technology (IT), wearable devices, telehealth and telemedicine, as well as emerging areas, such as the use of advanced computing sciences in big data, genomics and artificial intelligence^9,10^
e-Health	‘The use of information and communications technology (ICT) in support of health and health-related fields’^9^
Mobile health (mHealth)	‘The use of mobile wireless technologies for health’^9^
A digital health intervention	‘A discrete functionality of digital technology that is applied to achieve health objectives and is implemented within digital health applications and ICT systems, including communication channels such as text messages’^9^

Two recent systematic meta-reviews have identified the role and the effect of DHIs in the context of palliative care.^15,16^ Positive impacts have been reported on education, symptom management, information sharing, decision-making and communication in palliative care contexts. These interventions have been delivered most frequently *via* videoconferencing, electronic healthcare records, telephones or mobile phones, online interventions (websites and online courses) and social media.^16^ However, these reviews included studies that had been conducted in high-income settings without consideration of how such DHIs could be adapted to LMIC contexts.

The use of video consultation, e-health and telehealth in delivering palliative care have been examined in two systematic integrative reviews.^17–19^ Video consultations in palliative care have advantages for patients including avoiding the need for exhausting travel to hospitals or other healthcare facilities and for service delivery may enable the integration of general and specialized palliative care; for example, where different healthcare professionals and/or the patient and relative(s) are at different locations. Disadvantages include privacy and security issues, and lack of physical proximity.^18^ There have been very few formal outcome evaluations of mobile health (mHealth) in LMICs despite their potential to improve community health service delivery.^20^

Given the lack of evidence that relates directly to LMICs, this review aims to answer the question, ‘How have DHIs been adapted and utilized to provide palliative care in LMICs, and what factors influence the way that they are used?’

## Methods

### Design

A mixed-method systematic review was conducted following the Joanna Biggs Institute (JBI) convergent integrated approach for mixed-method reviews.^20^ This allows various methodologies, including non-experimental studies, to be analysed and synthesized by converting the quantitative data into narrative descriptions and interpretations to allow integration with qualitative data.

The review protocol was registered with Prospective register of systematic reviews (PROSPERO) (registration number: CRD42022331938) and reported in accordance with the Preferred Reporting Items for Systematic Reviews and Meta-Analysis (PRISMA) guidelines (Supplemental File 1).

The review had the following objectives:

1. To summarize the range and characteristics of DHIs used in the provision of palliative care for patients in LMICs.
2. To identify the factors that influence the implementation and utilization of different DHIs to provide palliative care for patients in LMICs.

### Inclusion and exclusion criteria

All the articles fulfilling the following criteria were included in this systematic review:

– Articles dealing with the use of DHIs in delivering palliative care or end-of-life care.
– Articles including patients in all ages, suffering from life-threatening diseases, in need of palliative care and/or their relatives/caregivers in all ages.
– Articles including healthcare professionals who are delivering palliative care for the people who are in need.
– Articles written on research-based qualitative, quantitative and mixed-methods studies (primary research).
– Articles published in English.

Publication date was not restricted. In summary, all studies focusing on DHIs for patients who need palliative care (adults/children) and their caregivers (patient and caregiver centred) in LMICs were considered; LMICs were decided based on the World Bank Definition of LMICs encompassing low-income economies, which were defined as those with a GNI per capita of $1045 or less, lower-middle-income economies, those with a GNI per capita between $1046 and $4095 and upper-middle-income economies, which were those with a GNI per capita between $4096 and $12,695.^3^ Studied were excluded if they were: secondary research, protocols, posters or conference proceedings, studies focusing only on health professionals and processes or tasks, including studies purely focusing on provider-to-provider communication, registries, vital events tracking, work-planning and scheduling, provider training and education, human resource management, supply chain management, financial transactions and incentives were excluded.

### Search strategy

An initial search of CINAHL and MEDLINE was carried out on 1st May 2022 to identify articles on the topic. The keywords/index terms used to describe the articles and the text words in the titles and abstracts of relevant articles informed the development of a complete search strategy. The search strategy, including all identified keywords and index terms, was adapted for each information source: MEDLINE, EMBASE, PsycINFO and CINAHL. The list of references of all the studies selected for critical appraisal was screened for additional studies.

Search terms were as follows: palliative care, end-of-life care, terminal care, digital health, e-health, telehealth, telemedicine, mobile health, mHealth, mobile phone, cell phone, mobile application, internet, video conferencing, low-income countries, middle-income countries, developing countries and names of all the low-/middle-income countries. The complete search strategies are provided in Supplemental Appendix 1. The full search was undertaken on 4th May 2022.

### Study selection

**Figure 1.**
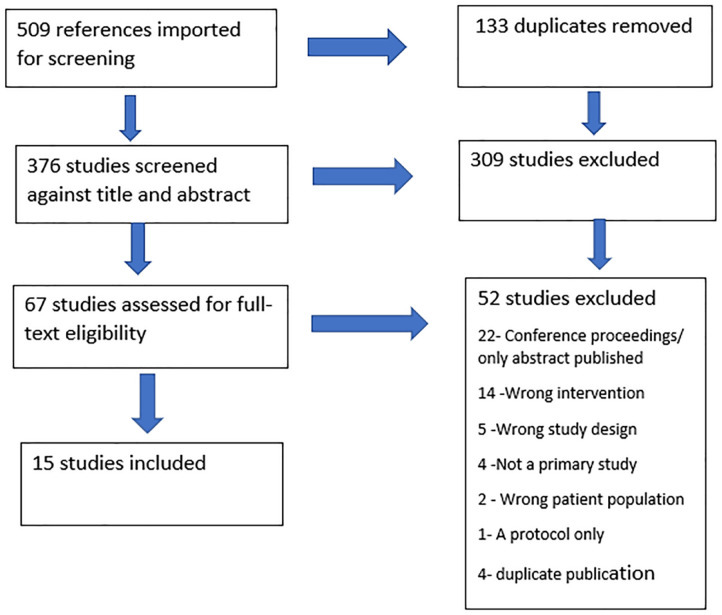
Preferred Reporting Items for Systematic Reviews and Meta-Analysis (PRISMA) flow diagram.

All identified citations were collated and uploaded into Endnote 20/2022 citation management system, and duplicates were removed. Each title and abstract retrieved were screened independently by two review authors (SD, KT and MJ) against the inclusion criteria. Then at least two review team members (SD, KT and MJ) retrieved the full text of any potentially eligible studies and independently assessed them for eligibility. Any disagreements regarding eligibility were discussed and resolved by consensus with a third team member (SM, JM and JD). Finally, the reasons for the exclusion of full-text studies that did not meet the inclusion criteria were recorded. The search results are reported and presented in a PRISMA flow diagram (Figure 1).

### Main outcome(s)

This review extracted data on factors affecting the implementation and use of DHIs including:

– Modes of DHIs that have been utilized to deliver palliative care in LMICs and the purposes of using those.
– Regional, organizational and individual levels factors seem to influence the implementation and utilization of different DHIs to provide palliative care for patients in LMICs.

### Data extraction (selection and coding)

– Two independent reviewers extracted the quantitative and qualitative data from each study included in the review. The following information were extracted into a bespoke data extraction table for the assessment of study quality and evidence synthesis.
– Study setting, publication year, study design, objectives, demographic details of study participants, intervention characteristics (content, format, mode of delivery) and outcomes (effectiveness, acceptability, feasibility, issues in implementation).
– For quantitative studies, extracted outcomes comprised of descriptive and/or inferential statistical results, whereas for the qualitative studies, data comprised themes or subthemes with corresponding illustrations.
– Any disagreements between the reviewers were resolved through discussions or with a third reviewer.

### Assessment of methodological quality

Included studies were assessed for methodological quality using the relevant standard JBI critical appraisal instruments^20^ by independent two review authors (SD, KT and MJ). Any disagreements between the review authors over the risk of bias in particular studies were resolved by involvement of a third review author where necessary (SM, JM and JD).

### Data transformation

The extracted quantitative data were converted into qualitized data facilitating the integration with data extracted from qualitative studies and the qualitative components of mixed-methods studies. This involved narrative interpretation to respond directly to the review question. Simply by qualitizing, ‘quantities’ were converted into declarative standalone sentences.

### Data synthesis and integration

A convergent integrated approach based on the JBI methodology for mixed-methods systematic reviews was adopted.^20^ This involved assembling the qualitized data with the qualitative data. Then, the assembled data were categorized and grouped based on similarity in meaning to produce a set of integrated findings in line with the research question. Firstly, the extracted qualitative data and qualitized data were assembled together. Then this pooled data were read through thoroughly to identify categories on the basis of similarity in meaning. A category consisted of two or more: qualitative data, ‘qualitized’ data or a combination of both. These categories were then aggregated to produce the overall finding(s) of the review.

## Results

The initial search yielded 509 published articles (97 from CINAHL, 13 from PsycINFO, 102 from MEDLINE and 297 from Embase). After removal of duplicates, 376 articles remained. These were screened based on title and abstracts, resulting in the exclusion of 309 articles. Consequently, 67 studies were assessed for full-text eligibility. Of these, 52 studies were excluded and 15 studies remained in the review for data extraction (Figure 1).

### Methodological quality

The studies represented a heterogeneous body of evidence, but all had clear aims and used appropriate methodology, and data were collected in a way that would address the research aims.

The outcomes of the appraisal process are presented in tables according to the type of study (Supplemental Appendix 2: Table numbered 1–4). Most quantitative studies and the quantitative component of the mixed-method studies were of good quality. No study was excluded based on its quality.

### Overview of studies

The studies included reports on the usage of DHIs in broad and varied healthcare settings (Table 2). Five mixed-methods studies,^21–25^ three qualitative studies,^8,26,27^ one quasi-experimental study,^28^ five quantitative studies (descriptive/analytical)^11,12,14,29,30^ and one case series^13^ published between 2013 and 2022, were included. A diverse range of methods were employed: interviews with healthcare providers, patients and caregivers,^8,22–24,26,27^ retrospective or prospective analysis of telemedicine records,^12,23^ healthcare professional surveys,^21,22,30^ a non-concurrent, controlled quasi-experimental study to evaluate a web-based intervention,^28^ prospective patient surveys^11^ and caregivers,^14,29^ a case series^13^ and the evaluation of a mobile app.^25^ Most of the studies were exploring patients living with advanced cancer,^11–14,22,25–28,30^ whereas a few were based on general palliative care patients.^23,24,29^ Characteristics of included studies are presented in Table 3.

**Table 2.** List of the country of origin of studies included in analysis.

Country of study	Studies included
India	11–13, 24
North and Sub-Saharan Africa	21, 27
Nigeria	8, 22
Uganda	26
Tanzania	25
Bangladesh	23
China	28
Mexico	10
Nepal	30
Columbia	29

**Table 3.** Summary of study characteristics of publications included in review.

Source	Country	Aim	Study design	Mode of data collection and sample	Type of DHI studied	Authors’ conclusion
Nwagwu *et al.*, 2013	Nigeria	To examine how ICTs are currently being used to share information by medical/paramedical team, families/relatives of the sick and other stakeholders for the purpose of ensuring adequate management of patients with advanced cancer at the University College Hospital, Ibadan, Nigeria’s premier and largest teaching hospital	Mixed-method study	Survey; using a questionnaire containing close and open-ended questions. *n* = 55 doctors Qualitative data: Semi-structured interviews using an interview guide. *n* = 6 doctors	Information communication technology	mHealth fit the working environment in the palliative care setting where nurses and doctors move around constantly and collaborate extensively.
Shabnam *et al.*, 2018	Bangladesh	To describe a 24/7 palliative care TCS; the development and the use of the service and the challenges experienced by the physicians delivering the consultations	Mixed-method study	1. A descriptive analysis of the documentation of calls from the *patients and their carers* (from January 2009 to August 2016) *n* = 4195 2. Semi-structured group interviews with *palliative care physicians*	24/7 palliative care TCS	The TCS offers a convenient way of delivering advice to palliative patients and their families in rural areas of Bangladesh, where face-to-face palliative care is unavailable
Karera *et al.*, 2022	Uganda	To explore health professionals’ perceptions about the role of mHealth in palliative cancer care, the challenges associated with its use and how it might support the advancement of palliative cancer care	Qualitative study	Secondary qualitative data analysis of semi-structured interviews among *healthcare professionals working at palliative care facilities* *n* = 20	mHealth	mHealth is considered advantageous for the provision of palliative cancer care. There is a need to adapt training to improve competencies of palliative care professionals in delivery of care that leverages digital technologies. In addition, consideration needs to be given to the maintenance of patient privacy and how to avoid inequities in access being worsened
Zhang *et al.*, 2019	China	To evaluate the feasibility and effects of WBLRP on anxiety, depression, self-transcendence, meaning in life and hope among cancer patients undergoing chemotherapy	Quantitative study	A non-concurrent, controlled quasi-experimental study among *advanced cancer patients* who are able to access the Internet *n* = 42 control group, *n* = 44 experimental group	Web-based intervention to improve the psychospiritual well-being of palliative patients	The innovative WBLRP is an effective non-pharmacological intervention in improving psychospiritual well-being of community-dwelling cancer patients. It could be integrated into transitional care for cancer patients
Chavarri-Guerra *et al.*, 2021	Mexico	To describe how a multidisciplinary supportive and palliative care programme was transformed into a telemedicine intervention for patients with advanced cancer during the COVID-19 pandemic	Quantitative study	Prospective online multiple-choice survey among patients with a recent diagnosis of advanced cancer *n* = 45	Multidisciplinary patient-navigator-led tele medicine supportive care programme	Implementing a supportive and palliative care telemedicine programme for patients with advanced cancer during the COVID-19 pandemic is feasible in low- and middle-income countries using readily available resources. Telemedicine represents an excellent method to maintain care continuity for patients with high symptom burden while limiting the risk of contagion for both patients and providers
Harding *et al.*, 2021	India, Uganda and Zimbabwe	To design a mobile phone application (app) to enable or improve communication between family caregivers, community caregivers and palliative care teams; to evaluate its acceptability, processes and mechanisms of action; and to propose refinements	Mixed-method study	Single group quantitative longitudinal data on application usage. Qualitative cross-sectional stakeholder data on the user experience. Purposive sampling at each site, *n* = 3 lay community caregivers, *n* = 9 family caregivers and *n* = 3 professionals	Mobile phone application for family or community caregivers to report nonurgent patient and family outcomes that appeared on a dashboard accessible by the clinical palliative care team	An outcomes-focused app and data dashboard are acceptable to caregivers and healthcare professionals. They are beneficial in identifying, monitoring and communicating patient outcomes and in allocating staff resource to those most in need
LeBaron *et al.*, 2021	Nepal	To design a mobile health application (‘app’) to scale-up implementation of existing locally developed pain management guidelines	Quantitative study	Cross-sectional study among healthcare professionals (nurses, physicians, pharmacists, trainees) Pencil-and-paper surveys *N* = 97	Smartphone/mobile phone	Healthcare professionals view cancer pain as an important symptom management concern, use smart phone apps frequently and are receptive to a mobile app to provide pain management support. Mobile apps must be informed by a clear understanding of contextual barriers to both cancer pain management and smart phone usage that are influenced by institutional resource and disciplinary differences
Biswas *et al.*, 2020	India	To assess how telemedicine can assist palliative medicine physicians to follow-up and manage cancer patients and the barriers that may be faced	Quantitative study	Prospective analysis of the telemedicine records of a total of 314 cancer patients	24 × 7 smartphone-based telemedicine service for cancer patients	Telemedicine is helpful for providing holistic integrated care to patients who are unable to visit hospitals regularly
Atreya *et al.*, 2020	India	To assess the changes in hospital-based palliative care in the COVID-19 pandemic and patient/caregiver’s perception about the provision of telehealth services	Quantitative study	Exploratory survey. A predesigned and pretested structured questionnaire was used for telephonic data collection among advanced cancer patients *n* = 50	Teleconsultation	Majority of the patients/caregivers felt that telemedicine was advantageous as an alternative tool for physical consultation
Biswas *et al.*, 2020	India	To explore the scope of telemedicine with audio-visual technology in patients with advanced cancer	A case series	3 patients receiving palliative care, diagnosed with metastatic stage 4 carcinoma	Smartphone-based application (WhatsApp) for the process of providing EOLC	Telemedicine service with an audio-visual facility seems to be an effective mode of providing EOLC to advanced cancer patients, particularly for patients who cannot commute to the hospices either due to their terminal illness or due to restrictions on movement during the pandemic period
Alvarez-Tobon *et al*., 2018	Columbia	To explore the demographic factors and level of knowledge related to information and communication technologies of potential users of a palliative care information system	Quantitative study	Cross-sectional survey using an interviewer administered questionnaire *n* = 35 patients, *n* = 39 caregivers	Palliative care information system using ICT	There is a need to focus on the needs of caregivers, and subjects facing challenges related to technology adoption
Allsop *et al.*, 2019	North Africa Sub-Saharan Africa (21 countries)	A survey of mobile phone use in the provision of palliative care services in the *African region* and priorities for future development	Mixed-method study	An online survey among PC professionals *n* = 51 responses from PC professional	Palliative care provision using mobile device	mHealth usage was noted across many of the 21 countries. As were the barriers to implementation of DHI’s. Further work is required to explore how DHIs can be optimized for use in PC provision
Morse *et al.*, 2021	Tanzania	To design and develop a web and mobile app to support outpatient symptom assessment and care coordination and control, with a focus on pain	Mixed-method study	Early pilot testing of the app among patients, caregivers and health professionals *n* = 10 patients and caregivers	mPCL prototype; a web and mobile app focused on symptom assessment and control	mPCL pilot testing demonstrated that all users were able to navigate the app and feedback suggested the mPCL has clinical utility. Further work is required to demonstrate effectiveness and sustainability of mPCL in symptom control of Tanzanian cancer patients
Nkhoma *et al.*, 2021	Nigeria, Uganda, Zimbabwe	To identify stakeholders’ information needs and the role of digital technologies to improve access to and delivery of palliative care for people with advanced cancer	Cross-sectional qualitative study in Sub-Saharan Africa	In-depth qualitative interviews conducted with patients, caregivers and healthcare professionals *n* = 62 advanced cancer patients, *n* = 48 informal caregivers, *n* = 59 healthcare professionals, *n* = 26 policymakers	Patient and caregivers use of technology to search and share information about cancer. Health professional’ use of technology to monitor and follow-up patients	Healthcare professionals supported the use of secure data systems, and patients welcome improved communication with providers. Preferences vary for how and when digital technologies should be utilized as part of palliative cancer care provision, including for increasing timely patient access to trained palliative care providers and the triaging of contact from patients
van Gurp *et al.*, 2015	Nigeria	Explores healthcare professionals’ concepts of a good death and how telemedicine technologies and services fit with current palliative care practice	Qualitative study	Three focus groups with Nigerian healthcare professionals interested in palliative care, unstructured interviews with key individuals for providing palliative care and representatives of telecom companies	Telemedicine	The addition of telemedicine to Nigeria’s palliative care practice faces challenges due to irregular bandwidth, poor network coverage and unstable power supply obstructing interactivity and access to information. However, a tele-education ‘lite’ scenario seems viable, with low-tech educational networks that build on non-synchronous online communication

DHI, digital health intervention; ICT, information and communications technology; mPCL, mobile-Palliative Care Link; TCS, telephone consultation service; WBLRP, WeChat-based life review programme; EOLC, End of life care; PC, palliative care.

### Range and characteristics of DHIs

Most studies described the use of either mHealth or telemedicine using a mobile phone, smartphone or landline as the mode of communication,^11–14,21–23,25,26,30^ and utilized phone calls, text messages, emails and mobile applications such as WhatsApp, Zoom, MSN Messenger or Skype for audiovisual consultations. Only one study from low-income,^26^ and two studies from Sub-Saharan African countries,^21,27^ which are a mix of LMICs were included to the review and all three studies have focused on use of mHealth/mobile phones by patients or healthcare professionals to communicate in between consultations through phone calls, text messages and a few participants have used WhatsApp and emails, especially for documents sharing.

Three studies evaluated the use of a newly designed app or platform to improve palliative care delivery: a multi country (India, Uganda and Zimbabwe), mixed-methods study discussed the design and evaluation of a novel mobile phone application for family or community caregivers to report nonurgent patient and family outcomes; these appeared on a dashboard accessible to the clinical palliative care team.^24^ Another quasi-experimental study conducted in China has piloted a WeChat-based life review programme for cancer patients, specifically designed to improve the psychospiritual well-being of palliative patients.^28^ A study conducted in Tanzania designed and evaluated ‘mobile-Palliative Care Link’ (mPCL), a web and mobile app to support advanced cancer patients, for outpatient symptom assessment and care coordination and control, with a focus on pain.^25^

The purposes of using DHIs were consistent across the studies. In most, patients and caregivers used mHealth/telemedicine/telephone consultation services to seek advice on managing symptoms, especially pain. Psychological symptoms such as anxiety and depression and other common symptoms of advanced cancer like vomiting, breathlessness and constipation were also mentioned.^11,12,23,26^ Patients and caregivers sought information on palliative care problems and needs for support. This included queries relating to follow-up visits, hospital admission, home care or restocking their medications and other related supportive care services.^12,23^ Two articles reported patient and caregiver education as the main purpose of the mHealth intervention.^21,30^ In most cases, teleconsultation enabled palliative care physicians to provide advice on pain and other symptom management, psychological care and nutritional counselling.^11,12,23^ Furthermore, they provided information and contact details of other palliative care support teams for home visits and other needs.^11,12^

Among the reviewed articles, there were no studies focusing only the palliative care needs of children specifically. Though, one study (23) has analysed the telemedicine records, which included the caregivers of children with palliative care needs, they have not presented the results separately for children and adults. Considering the limited data availability, it is difficult to make distinction between DHIs used in the care of children and adults.

The use of smartphone-based applications (including WhatsApp) have been evaluated in the provision of palliative care^13^ for reporting patient and family outcomes to a dashboard that was accessible by the clinical palliative care team,^24^ and also to improve the psychospiritual well-being of patients.^28^ A survey of cancer care institutions in Nepal explored physicians’ opinions on using mHealth for pain management and reported ‘prescribing/giving opioid medications’, ‘prescribing/giving non-opioid medications’ and ‘sharing information with learning from other health care providers’ as the most frequent ways that mobile app would be helpful to them.^30^

A survey describing mHealth in 21 African countries located in North Africa and Sub-Saharan Africa identified two broad categories: patient-centred tasks and health professional/process tasks. The former included patient education and behaviour change communication, the use of sensors and point of care diagnostics, data collection and reporting and electronic decision support.^21^

### DHIs and the impact of COVID-19

The COVID-19 pandemic brought new challenges in the delivery of palliative care in LMICs, including increased need for services and a need to deliver care remotely to reduce the risk of infection for patients and carers. Increased use of teleconsultations was associated with a drop-in footfall at the care facility.^14^ The use of smartphones for patients receiving palliative care during the pandemic was associated with a high level of caregiver satisfaction. The integration of video calls alongside conventional phone calls and texts was a powerful combination.^12^ A multidisciplinary patient-navigator-led telemedicine supportive care programme conducted in Mexico during the pandemic to deliver palliative care demonstrated the feasibility of providing palliative care interventions using telemedicine in resource-limited settings.^11^

### Benefits in utilizing DHIs in delivering palliative care in LMICs

Four of the reviewed studies focused on the benefits of using DHIs in delivering palliative care, while most of the other studies also reported that it is advantageous, beneficial or acceptable in the settings studied. Healthcare providers identified that DHIs make their work more accessible, enabling the provision of more effective and efficient care and enabling patients to be managed in distant places.^14,22,24,26^ Study participants felt that this allowed healthcare cost reductions and improved patient-provider relationships.^26^

For patients and caregivers, the benefits of using DHIs included increased access to care, reduced travel time, reduced risk of healthcare-associated infections, reduced unnecessary visits to outpatient departments and reduced hospital admissions. Patients also reported benefits in the provision of firsthand information and feeling reassured, cared for and comforted.^14,26^ Furthermore, patients and families felt that it was easy to contact palliative care services to get their opinion when it was needed, irrespective of their location and transport challenges. The use of DHIs prevented the cost and stress associated with travelling and long waiting times at the clinic.^27^

There was evidence that DHIs had the potential to improve both access and convenience in delivering palliative care in LMICs. For example, an evaluation of a 24-h palliative care telephone advice service in Bangladesh found that it enabled advice-giving to palliative care patients in rural areas, which received very positive feedback from patients.^23^ A quasi-experimental study conducted in China to evaluate the feasibility and effects of a WeChat-based life review programme showed a statistically significant impact in reducing anxiety in community-dwelling cancer patients.^28^ Evaluation of an mHealth application to improve palliative care at home in India, Uganda and Zimbabwe revealed high acceptability with healthcare professionals reporting benefits in identifying and monitoring patient outcomes, enabling re-allocation of staff resources to those most in need.^24^ A study conducted to design and test an app to improve symptom control and information exchange among specialists and local health workers treating Tanzanian cancer patients suggested this was acceptable, and early pilot testing showed it to be usable and feasible.^25^

### Barriers to implementation

We identified four themes characterizing the main barriers to implementing DHI in LMICs: (1) resource constraints; (2) literacy, training and skills; (3) governance, operational and communication issues and (4) technical issues.

#### Resource constraints

The affordability of devices such as smartphones used in mHealth or in delivering specific interventions through mobile phone applications may be a challenge, especially for patients.^21,22,27^ In addition, for DHIs which require internet access, the further cost of data access is a challenge for both patients and healthcare professionals.^26,27,30^ Cheaper methods of communicating, such as WhatsApp, are not consistently available on cheaper phone deals.^21^ At an institutional level, limited access to both the expertise and hardware required for implementing DHIs is also a barrier.^21,26,30^

#### Literacy, training and skills

Low levels of literacy, IT skills and limited experience or awareness of communicating *via* DHIs is frequently described as a patient-reported barrier.^11,23,24^ The possibility of misinterpretation or misunderstanding arising when communicating using mHealth and language barriers are further relevant concerns.^26^ Healthcare providers also recognize lack of training in DHIs as a barrier to their use.^23,26^ At the institutional level, limited access to professional expertise required for mHealth use, limited expertise to set up and use new digital technologies and lack of computer literacy in healthcare teams have been identified as constraints.^21^

#### Governance, operational and communication issues

Healthcare providers, especially physicians and nurses, identified governance, operational and communication issues while using DHIs. Among them, issues of data security and confidentiality were major concerns.^26^ Crowded households can be a barrier to discussing personal issues.^11^ Sharing phones can also risk breaches of confidentiality.^26^ Recommended solutions include using password protected phones, as well as maintaining end-to-end encryption for mHealth.^11^ Inconsistent access to patient records during remote consultations and a lack of familiarity between healthcare professionals and patients were also challenges.^23^ Local regulatory issues, such as nurses not being allowed to use a mobile phone at work and smartphones not being allowed in the presence of patients, were further barriers to implementation.^30^ In addition, limited administrative support to take on additional tasks associated with mHealth approaches and governance constraints arising from resistance to adoption at national or local level have also been identified.^21^

#### Technical issues

Technical issues include patients and caregivers lacking access to the internet because of unreliable network connectivity issues, poor network coverage and limited internet in some countries.^8,21,24,26,29^ In addition, limited electricity access, problems with electricity supply or unstable power supply obstructing interactivity and access to modern IT was a further challenge.^8,21,26,27^ This was particularly noted as a barrier for video conferencing due to interruptions and poor-quality video/audio associated with connectivity issues.^11^

## Discussion

This systematic review has bought together 15 methodologically heterogeneous studies that explore DHIs used in diverse LMIC settings to support the delivery of palliative care. The review’s findings indicate that mHealth is growing in prominence as a DHI for palliative care and is mainly used by patients and caregivers to seek advice on managing symptoms. Several smartphone-based apps and web-based interventions have been piloted and reported as providing positive impact on patients’ and caregivers’ well-being. Although the findings suggest that DHIs have the potential to improve the delivery of palliative care in LMICs, barriers to their adoption by services include resource constraints, healthcare professional proficiency, governance and technical concerns. Common barriers for patients and caregivers include access to smartphones and the internet, digital literacy, stability of internet connection, poor video and audio quality and privacy issues.

Mobile phone ownership is projected to grow to half of the Sub-Saharan African population by the year 2025.^31,32^ This has potential positive implications for the future implementation of DHIs for palliative care,^32^ although more needs to be done to understand how this improved accessibility increases the capacity for DHI provision in palliative care delivery. For example, there are gaps in palliative care education in LMICs, including the African region,^33^ and clinicians have concerns about the lack of mHealth in their current training programmes.^26^ It is critical to consider the training needs of both patients and carers and clinicians in the use of DHIs. There is also a larger question about the social and technological infrastructure in LMICs and the availability of resources that can support DHI delivery in palliative care. This is a challenge that many LMICs face and an improved infrastructure that can provide consistent and reliable DHI will not only improve palliative care but also healthcare overall.

Although the findings of this review suggest that patients and carers feel there are benefits to the use of telemedicine, there is still evidence that patients often prefer seeing a healthcare professional face to face rather than digitally, especially when there are psychological concerns to discuss.^23^ It may therefore be most appropriate to consider the use of DHIs alongside the provision of face-to-face consultations rather than as a substitute or replacement.^17^

### Comparison with existing literature

The findings of two recent systematic reviews^15,16^ of the use of DHIs in the palliative care setting substantiate our findings, with regard to its applicability and its range of use, neither attempted to contextualize DHIs in LMICs specifically. This is important to highlight as the social and economic contexts are key determinants in both the quality and robustness of healthcare infrastructure. Our review complements this earlier work by highlighting the barriers to implementation of DHIs in LMICs. LMICs contend with a unique set of challenges as the social and economic infrastructure may limit healthcare provision. This is where our work can be of most benefit, as by highlighting these issues it will provide further clarity and guidance on how DHIs could be implemented in the palliative care setting within LMICs.

### Strengths and limitations

This review investigates an important area of palliative care practice and one that requires more work and consideration. A strength is that it followed a rigorous review method (JBI), with a dual screening process for inclusion of studies, data extraction and quality assessment. The inclusion of both qualitative and quantitative studies allowed a comprehensive understanding of the issues that influence the adoption and use of DHIs in LMICs to emerge.

Findings from this review should be interpreted cautiously as the literature considered in the review was quite limited. As the review was limited to published articles, potentially relevant evidence in the grey literature was not covered. The included studies were of variable quality, although none were excluded based on quality assessment due to the limited evidence base available for the review. Furthermore, some studies that met the inclusion criteria could not be included as the full text was not published.

## Conclusion

This review has found that the use of DHIs in delivering palliative care in LMICs is feasible and could be beneficial. However, the barriers at the interpersonal and healthcare system level are significant, and careful consideration and planning is needed to address them. Further work is required to explore how existing DHIs might be developed to be aligned with priority areas for palliative care development in LMICs. The use of DHIs in areas where face-to-face palliative care services are not accessible due to rural and remote geography requires further investigation. Plans to implement DHIs should be accompanied by training in their use for healthcare professionals as well as patients and their carers.

## Supplemental Material

sj-docx-1-pcr-10.1177_26323524241236965 – Supplemental material for A mixed-methods systematic review investigating the use of digital health interventions to provide palliative and end-of-life care for patients in low- and middle-income countries Supplemental material, sj-docx-1-pcr-10.1177_26323524241236965 for A mixed-methods systematic review investigating the use of digital health interventions to provide palliative and end-of-life care for patients in low- and middle-income countries by Weerasingha Navarathnage Sachintha Dilhani, Sarah Mitchell, Jeremy Dale, Kavanbir Toor, Mikail Javaid and John I. MacArtney in Palliative Care and Social Practice

sj-docx-2-pcr-10.1177_26323524241236965 – Supplemental material for A mixed-methods systematic review investigating the use of digital health interventions to provide palliative and end-of-life care for patients in low- and middle-income countries Supplemental material, sj-docx-2-pcr-10.1177_26323524241236965 for A mixed-methods systematic review investigating the use of digital health interventions to provide palliative and end-of-life care for patients in low- and middle-income countries by Weerasingha Navarathnage Sachintha Dilhani, Sarah Mitchell, Jeremy Dale, Kavanbir Toor, Mikail Javaid and John I. MacArtney in Palliative Care and Social Practice

## Declarations

**Ethics approval and consent to participate:** Not applicable.

**Consent for publication:** Not applicable.

**Availability of data and materials:** Sharing of data files is not necessary or applicable as ‘all data appear in the article as appendices’.
