# Supplementary material for: A mixed-methods systematic review investigating the use of digital health interventions to provide palliative and end-of-life care for patients in low- and middle-income countries
Source: Palliat Care Soc Pract. 2024 Apr 12;18:26323524241236965. doi: 10.1177/26323524241236965 (PMC11010586; doi:10.1177/26323524241236965)
Supplement: sj-docx-1-pcr-10.1177_26323524241236965 – Supplemental material for A mixed-methods systematic review investigating the use of digital health interventions to provide palliative and end-of-life care for patients in low- and middle-income countries [file sj-docx-1-pcr-10.1177_26323524241236965.docx]

| Key word  **Appendix 1: Search Strategy** | Alternative | Alternative | Alternative | Alternative |
| --- | --- | --- | --- | --- |
| Palliative care | End of life care | Terminal care |  |  |
| Digital health | e health or electronic health | telehealth / tele medicine | mobile health/ m health/mobile phone /Cell phone/ mobile application | Internet / video conferencing |
| Low and middle income countries | Developing countries | BRICS /BRIC | BRICS or BRIC or Africa or "north africa" or "northern Africa" or "Africa South of the Sahara" or "Central Africa" or "east Africa" or "Eastern Africa" or "Southern Africa" or "Western Asia" or "central Asia" or "southeastern Asia" or "Caribbean" or "West Indies" or "South America" or "Latin America" or "Central America" or Afghanistan or Albania or Algeria or "American Samoa" or Angola or "Antigua and Barbuda" or Argentina or Armenia or Azerbaijan or Bahrain or Bangladesh or Barbados or Benin or Belarus or Belize or Bhutan or Bolivia or Bosnia-Herzegovina or Botswana or Brazil or Bulgaria or "Burkina Faso" or Burundi or Cambodia or Cameroon or "Cape Verde" or "Central African Republic" or Chad or Chile or China or Colombia or Comoros or Congo or "Costa Rica" or "Cote d'Ivoire" or Croatia or Cuba or Cyprus or Czechoslovakia or "Czech Republic" or Slovakia or Djibouti or "Democratic Republic of the Congo" or Dominica or "Dominican Republic" or "East Timor" or Ecuador or Egypt or "El Salvador" or Eritrea or Estonia or Ethiopia or Fiji or Gabon or Gambia or "Georgia" or Ghana or Greece or Grenada or Guatemala or Guinea or "Guinea-Bissau" or Guam or Guyana or Haiti or Honduras or Hungary or India or Indonesia or Iran or Iraq or Jamaica or Jordan or Kazakhstan or Kenya or Korea or Kosovo or Kyrgyzstan or Laos or Latvia or Lebanon or Lesotho or Liberia or Libya or Lithuania or Macedonia or Madagascar or Malaysia or Malawi or Mali or Malta or Mauritania or Mauritius or Mexico or Micronesia or "Middle East" or Moldova or Mongolia or Montenegro or Morocco or Mozambique or Myanmar or Namibia or Nepal or "Netherlands Antilles" or "New Caledonia" or Nicaragua or Niger or Nigeria or Oman or Pakistan or Palau or Panama or "Papua New Guinea" or Paraguay or Peru or Philippines or Poland or Portugal or "Puerto Rico" or Romania or Russia or Rwanda or "Saint Kitts and Nevis" or "Saint Lucia" or "Saint Vincent and the Grenadines" or Samoa or "Saudi Arabia" or Senegal or Serbia or Montenegro or Seychelles or "Sierra Leone" or Slovenia or "Sri Lanka" or Somalia or "South Africa" or Sudan or Suriname or Swaziland or Syria or Tajikistan or Tanzania or Thailand or Togo or Tonga or "Trinidad and Tobago" or Tunisia or Turkey or Turkmenistan or Uganda or Ukraine or Uruguay or USSR or Uzbekistan or Vanuatu or Venezuela or Vietnam or Yemen or Yugoslavia or Zambia or Zimbabwe) |  |
|  |  |  |  |  |

**A mixed methods systematic review investigating the feasibility of digital health interventions to provide palliative and end of life care for patients in low- and middle-income countries**

1."palliative care" or "end of life care" or "terminal care"

2. “digital health” or “e health” or “electronic health” or “telehealth” or “tele medicine” or “mobile health” or “m health” or “mobile phone” or “Cell phone” or “mobile application” or “Internet” or “video conferencing”

3.“low income countries” or “middle income countries” or “developing countries” or BRICS or BRIC or Africa or "north africa" or "northern Africa" or "Africa South of the Sahara" or "Central Africa" or "east Africa" or "Eastern Africa" or "Southern Africa" or "Western Asia" or "central Asia" or "southeastern Asia" or "Caribbean" or "West Indies" or "South America" or "Latin America" or "Central America" or Afghanistan or Albania or Algeria or "American Samoa" or Angola or "Antigua and Barbuda" or Argentina or Armenia or Azerbaijan or Bahrain or Bangladesh or Barbados or Benin or Belarus or Belize or Bhutan or Bolivia or Bosnia-Herzegovina or Botswana or Brazil or Bulgaria or "Burkina Faso" or Burundi or Cambodia or Cameroon or "Cape Verde" or "Central African Republic" or Chad or Chile or China or Colombia or Comoros or Congo or "Costa Rica" or "Cote d'Ivoire" or Croatia or Cuba or Cyprus or Czechoslovakia or "Czech Republic" or Slovakia or Djibouti or "Democratic Republic of the Congo" or Dominica or "Dominican Republic" or "East Timor" or Ecuador or Egypt or "El Salvador" or Eritrea or Estonia or Ethiopia or Fiji or Gabon or Gambia or "Georgia" or Ghana or Greece or Grenada or Guatemala or Guinea or "Guinea-Bissau" or Guam or Guyana or Haiti or Honduras or Hungary or India or Indonesia or Iran or Iraq or Jamaica or Jordan or Kazakhstan or Kenya or Korea or Kosovo or Kyrgyzstan or Laos or Latvia or Lebanon or Lesotho or Liberia or Libya or Lithuania or Macedonia or Madagascar or Malaysia or Malawi or Mali or Malta or Mauritania or Mauritius or Mexico or Micronesia or "Middle East" or Moldova or Mongolia or Montenegro or Morocco or Mozambique or Myanmar or Namibia or Nepal or "Netherlands Antilles" or "New Caledonia" or Nicaragua or Niger or Nigeria or Oman or Pakistan or Palau or Panama or "Papua New Guinea" or Paraguay or Peru or Philippines or Poland or Portugal or "Puerto Rico" or Romania or Russia or Rwanda or "Saint Kitts and Nevis" or "Saint Lucia" or "Saint Vincent and the Grenadines" or Samoa or "Saudi Arabia" or Senegal or Serbia or Montenegro or Seychelles or "Sierra Leone" or Slovenia or "Sri Lanka" or Somalia or "South Africa" or Sudan or Suriname or Swaziland or Syria or Tajikistan or Tanzania or Thailand or Togo or Tonga or "Trinidad and Tobago" or Tunisia or Turkey or Turkmenistan or Uganda or Ukraine or Uruguay or USSR or Uzbekistan or Vanuatu or Venezuela or Vietnam or Yemen or Yugoslavia or Zambia or Zimbabwe

1 and 2 and 3
